# Improving Breast Surgery Outcomes Through Alternative Therapy: A Systematic Review

**DOI:** 10.7759/cureus.23443

**Published:** 2022-03-24

**Authors:** Yasmine Abushukur, Camilla Cascardo, Yousef Ibrahim, Fayven Teklehaimanot, Rebecca Knackstedt

**Affiliations:** 1 Medicine, Oakland University William Beaumont School of Medicine, Royal Oak, USA; 2 Medicine, Oakland University William Beaumont School of Medicine, Roha, USA; 3 Medicine, Michigan State University College of Osteopathic Medicine, Detroit, USA; 4 Plastic Surgery, Cleveland Clinic, Cleveland, USA

**Keywords:** breast and endocrine surgery, integrative medicine, breast, breast cancer, complementary medicine, alternative medicine

## Abstract

Enhanced recovery after surgery (ERAS) protocols are the current standard of care when it comes to improving post-surgical outcomes in breast cancer patients. Compliance with all protocol items is required in order for patients to experience significant benefits. Given that the ERAS protocols involve numerous medications which each have unique side effect profiles and medication interactions, this is often difficult to accomplish. Additionally, breast cancer patients are often left with a large psychological burden, which ERAS protocols fail to address. This review aims to determine the role that alternative therapies can play in improving both the emotional and physical strains patients experience during the post-operative stage of recovery. A PubMed search was conducted using the following search terms (“alternative medicine” or “complementary medicine” or “integrative medicine” or “holistic medicine” or “natural medicine" or “mediation” or “aromatherapy” or “music” or “art” or “reiki” or “massage”) and (“surgery”) and (“pain”). Studies selected for this review include articles published or translated in English that addressed alternative medical interventions affecting pre-, peri-, or post-operative outcomes in breast biopsies, surgeries, or breast-related procedures. Eighteen articles fit the inclusion criteria, with seven addressing music, five addressing meditation, yoga, and guided imagery, five addressing massage, one addressing myofascial release, four addressing aromatherapy, two addressing acupuncture, and three addressing hypnosis. Most forms of alternative therapies offered some benefit to patients following breast-related procedures, many resulting in improvements in post-operative outcomes including pain, fatigue, energy, stress, anxiety, mood, and depression. The reviewed studies demonstrated numerous benefits to integrating alternative medicine into standardized therapy to improve postoperative outcomes. Most studies analyzed did not include placebo controls as including proper placebos was often not feasible. Future studies with larger sample sizes are needed to better quantify the benefits patients receive from these noninvasive, low-risk complementary therapies.

## Introduction and background

Breast cancer is one of the most common cancers amongst women in the United States, second only to skin cancer [1]. Each year, about 255,000 new cases of breast cancer are diagnosed in the United States. Additionally, about 100,000 women go on to have some form of mastectomy each year either prophylactically or as a definitive treatment [2]. Unfortunately, these procedures can be very burdensome in terms of post-operative pain and emotional burden for patients. Breast biopsies alone have been shown to contribute to anxiety and depression in women both before and after the procedure due to fear of the procedure itself as well as fear of a potential cancer diagnosis [3]. Therefore, pain management and mental health support are crucial in achieving an optimal recovery [4].

The American Society of Breast Surgeons (ASBrS) has compiled a workgroup to encourage breast surgeons to consider the use of non-opioid alternatives to combat post-operative pain control and mitigate the current opioid crisis. This workgroup has recommended the use of a multidisciplinary approach in combination with standardized quantities of narcotics. It also strongly supports the use of the enhanced recovery after surgery (ERAS) protocol [5]. ERAS protocols have successfully implemented evidence-based practices to reduce post-operative pain, nausea, vomiting, opioid use, and length of hospital stay [6]. They aim to optimize patient outcomes during the pre-surgical, surgical, and post-surgical intervals. This can be accomplished through patient education and pre-surgical counseling, early transitions to oral pain medications post-operatively, and expedited post-procedure mobilization, for example [7]. Although ERAS protocols have been proven to lower both recovery time and post-operative complications in a cost-effective manner, compliance to all protocol items can be difficult to accomplish [8-10].

Gillis et al. conducted a patient-led narrative style study assessing the ERAS protocol patient experience, which brought to light several unaddressed patient needs. The success of ERAS protocols lies in the physical components of surgical recovery; however, these protocols lack to address patients’ emotional needs. In Gillis et al.’s study, patients felt ill-equipped to resolve stressors on their own during the pre-surgical phase and they were not informed of available community resources by their healthcare providers [11]. Importantly, patients with a higher psychological burden prior to undergoing a mastectomy faced poorer post-surgical outcomes, including a higher risk of complications, prolonged hospital stays, and increased costs of care [12].

Some women with breast cancer occasionally desire to utilize alternative remedies as a complement to traditional medications [13]. Many women who chose to use complementary therapies cited experiencing greater motivation to heal and an improved sense of control over their health [14]. The therapies utilized included massage, meditation, hypnosis, music, myofascial release, aromatherapy, guided imagery, and electro-puncture. The goal of this paper is to provide a systematic review of the adjunctive therapies that have been implemented to augment post-operative recovery in breast surgery patients.

## Review

Methods

A systematic literature review was conducted in order to determine what non-pharmacologic treatments could be used to optimize breast cancer patient care pre-, peri-, or post-operatively. The search terms were as follows: (“alternative medicine” or “complementary medicine” or “integrative medicine” or “holistic medicine” or “natural medicine” or “mediation” or “aromatherapy” or “music” or “art” or “reiki” or “massage”) and (“surgery”) and (“pain”). All articles were uploaded from PubMed into COVIDENCE, an online software program used for the production of systematic reviews. Once imported into COVIDENCE, all abstracts underwent initial screening by members of the review team. Studies included in this review required at least one alternative medicine intervention and a description of a pre-, post-, or peri-operative surgical outcome (pain, opioid requirements, infection rate, wound healing, length of stay, hospital readmission, etc.). All systematic reviews, literature reviews, meta-analyses, opinion pieces, non-English, non-human, chronic pain studies, and studies on non-surgical screening techniques (ex: ultrasound, mammography, etc.) were excluded. Articles with a focus on medications including vitamins, herbs, and supplements were also excluded from this study. Following abstract screenings, the full text of each article was screened using the predetermined inclusion/exclusion criteria, and qualitative data was extracted from all articles which met this criterion. Additionally, the references of select articles were screened in order to identify and include any additional relevant studies.

Results

**Figure 1:**
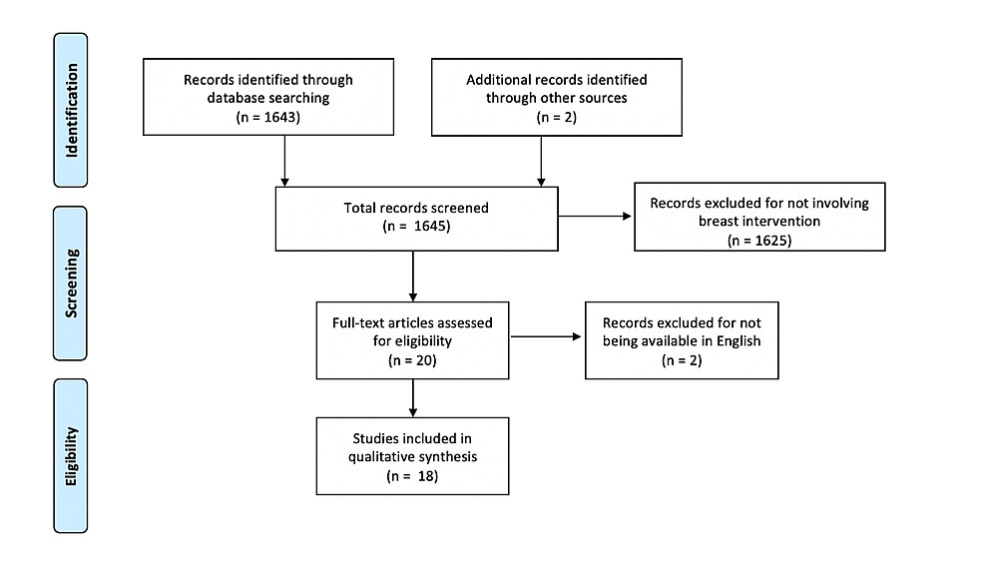
Preferred reporting items for systematic reviews and meta-analyses (PRISMA) algorithm adopted in the article selection process

The original search identified 1,645 total papers related to surgery and holistic medical interventions. Of that, 1,625 studies were excluded because they lacked a surgical intervention on the breast. Of the 20 remaining papers, two were excluded from being non-English studies. Thus, 18 met the criteria for review (Figure 1). The alternative medicine interventions identified in this review include massage therapy, meditation, hypnosis, music, myofascial release, aromatherapy, guided imagery, acupuncture, and electro-puncture.

Massage/Reflexology

Under the overarching term massage, several studies in this review covered subtypes of massage including reflexology, Swedish massage, and acupressure. Reflexology has been used since 2330 BCE to alleviate pain and stress [15,16]. The three main theories behind its efficacy are energy channeling, the breaking down of lactic acid build up and the utilization of the neuromatrix to prevent the transmission of pain [16]. Varying pressures are applied to different areas of the foot. The area that corresponds to the breast spans the distal, dorsal aspect of the foot, proximal to the phalanges. Acupressure is a similar form of therapy that utilizes manual pressure, commonly performed with the fingertips to specific points in the body with the goal of releasing muscle tension.

Three papers utilized foot reflexology as a form of massage. Ucuzal et al. specifically used reflexology in the experimental group in addition to analgesic therapy, while the control group was provided with analgesic therapy alone. This trial demonstrated a significant improvement in pain compared to the control group following reflexology as determined by the Short-Form McGill Pain Questionnaire [17].

The two other studies compared the effectiveness of massage therapy alone to a massage therapy in combination with other interventions. Dilaveri et al. studied breast cancer patients undergoing reconstructive surgery who were evaluated post operatively using visual analog scores (VAS) to determine pain, mood, energy, relaxation, insomnia, stress, anxiety, alertness, fatigue, and tension after massage alone or with the addition of acupuncture. The massage techniques utilized included foot reflexology and Swedish massage. Using the VAS scores, Dilaveri et al. determined that stress and anxiety decreased significantly, while relaxation increased in both treatment groups. Although both groups showed improvement, the massage-only group experienced a greater effect in all metrics [18]. Similarly, Dion et al. concluded that while massage and massage with the addition of meditation individually demonstrated a benefit, the addition of meditation to massage resulted in no additional change to VAS scores when compared to the massage-only group [19].

Thus, the use of massage alone as an adjunctive therapy to patients following breast procedures has shown to be beneficial and can reduce numerous post-operative symptoms including pain and anxiety.

Myofascial Release

Fascia is the connective tissue that encases various structures in the human body. It has tremendous tensile strength and any disruption in the fascial planes can cause dysfunction, pain, and discomfort. Myofascial release is the practice of placing direct pressure on the restricted fascia until a moment of release is felt. Serra-Ano et al. studied the effectiveness of myofascial release compared to placebo manual lymphatic drainage in patients who underwent breast cancer surgery. Fascial manipulation improved the range of motion in the shoulder determined by measuring the active angular reach via a goniometer. Pain severity, measured with VAS scores, was also decreased in patients undergoing myofascial manipulation with greater significance than those undergoing placebo manual lymphatic drainage [20].

Music

Music was first reported to aid in surgical procedures in 1914 [21]. Music can be incorporated into the pre-, peri-, and post-operative time periods with the goals of reducing pain, anxiety, and improving overall patient comfort. The pathophysiology behind its benefit is thought to be due to its ability to attenuate the neuroendocrine stress response to surgery [22]. The genre, dynamics, and duration of the music played is patient dependent, as long as it is used in an appropriate clinical setting.

Six studies evaluated the effect of music on breast-related surgical procedures. Tellez et al. aimed to determine the effect of music on breast biopsies. By analyzing VAS scores, the study concluded that when compared to a standard breast biopsy, the pre- or post-operative addition of music reduced stress, pain, and anxiety [3].

Deng et al. compared the effects of music and the combination of music and aromatherapy in the peri-operative treatment of breast cancer. Patients treated with music therapy demonstrated a reduction in pain intensity and anxiety when compared to standard therapy alone and the combination of music and aromatherapy demonstrated an even greater decrease in pain intensity and anxiety [23].

Soo et al. investigated the impact of relaxing music played during an image-guided core-needle breast biopsy. Using multiple questionnaires, there was a demonstrated reduction in anxiety, fatigue, and pain when compared to the standard care control [24]. Wren et al. studied the effects of music in patients undergoing breast biopsy or breast cancer surgery. There was a significant reduction in pain in those listening to pre-, peri-, and post-operative music compared to the control group [25]. Li et al. similarly demonstrated a reduction in pain for women undergoing radical mastectomy with music therapy [26]. Thus, the addition of music therapy in breast-related surgical procedures has been shown in numerous studies to improve anxiety, fatigue, pain, and stress in the post-operative period.

Aromatherapy

Aromatherapy is the use of essential oils that come from seeds, stems, leaves, needles, petals, flowers, rinds and fruits, woods and resins, roots and rhizomes, and grasses for medical purposes [27]. Four papers explored the use of aromatherapy in breast-related procedures. Chao et al. demonstrated that aromatherapy resulted in a decrease in pain, anxiety and levels of IL-6 and HMGB-1 compared to standard therapy. While the combination of aromatherapy and music therapy was superior to either intervention alone in reducing pain and anxiety, there was no significant difference between music-only and aromatherapy-only interventions [23].

Three papers explored the effect of lavender aromatherapy. Kim et al. investigated the addition of lavender to post-operative oxygen therapy. There was no difference in narcotic requirements or objective pain scores between control and intervention; however, patients in the lavender group reported a higher satisfaction rate with pain control than control patients [28]. Franco et al. explored the addition of either lavender fleur oil (LFO) or unscented oil (UO) aromatherapy in the care of breast surgery patients. The study demonstrated that the addition of LFO significantly decreased post-operative anxiety relative to the control group [29]. Shammas et al. examined the effects of lavender oil on post-operative breast cancer outcomes but found no significant differences in peri-operative depression and anxiety scores, pain scores, or sleep scores between the control and intervention groups [30]. 

Overall, while there were no adverse effects of aromatherapy documented, the evidence supporting the benefits of aromatherapy was mixed in the studies analyzed.

Guided Imagery, Hypnosis, and Meditation

Guided imagery is a relaxation technique, also known as visualization, that involves creating specific conscious experiences, such as imagining oneself on a beach, without the use of external stimuli [31]. When fully immersed in this technique, one can truly perceive the event created by their thoughts [32,33]. According to the United States National Center for Health Statistics, approximately five million adults report using guided imagery to reduce stress and address health-specific complaints [32]. Guided imagery is often used in combination with mediation, hypnosis, and other relaxation exercises as all of these focus on profound thought formation [34,35].

Kwekkeboom et al. tested the efficacy of guided imagery in reducing post-operative pain in women with breast and gynecologic cancers. Participants who underwent breast surgery completed guided imagery tasks and returned pain diaries 48 hours after discharge. Though this study reported a high percentage of patients opting to implement nonpharmacological pain management strategies, pain-related intensity and distress remained similar among patients who used analgesics alone and those who used an analgesic in combination with a nonpharmacologic intervention, such as guided imagery, re-positioning, heat, music, or meditation [36].

Three papers examined the impact of meditation as an intervention, one in patients undergoing autologous tissue reconstruction and two in patients undergoing breast biopsies. Using various scales, both Soo et al. and Wren et al. found that guided meditation reduced pain and anxiety about both breast surgery and the potential cancer diagnosis. Additionally, Wren et al. demonstrated a significant improvement in self-compassion and heart rate over time compared to the control group. The papers differed in that Soo et al. saw a significant reduction in fatigue scores following biopsy, while Wren et al. demonstrated no difference in fatigue compared to controls [24].

Dion et al. analyzed patients who underwent autologous tissue reconstruction and were treated with either massage or massage in combination with mediation. There was no difference in stress, anxiety, relaxation, insomnia, alertness, fatigue, tension, pain, mood, and energy between the two cohorts [19].

In two studies comparing hypnosis to a control group, both demonstrated significant psychological benefits. Schnur et al. investigated the effects of presurgical hypnosis on psychological stress in patients undergoing excisional breast biopsy. Prior to surgery, the hypnosis group had significantly improved relaxation and anxiety and decreased emotional upset and depression compared to the control group [37].

Montgomery et al. investigated the effects of adding a 15-minute hypnotic session prior to a breast-related surgical intervention. Patients receiving required less propofol and lidocaine during the intervention and had decreased post-operative pain, discomfort, fatigue, and nausea [38].

Overall, in terms of pain management, patients who took analgesics experienced similar outcomes to those using guided imagery in combination with analgesics. However, hypnosis and meditation both independently demonstrated significant pain reduction and psychological benefits for patients.

Yoga Therapy

Yoga is a practice that combines a sequence of postures with purposeful breathing and heightened self-awareness in order to achieve a state of relaxation and increased awareness of the mind, body, and spirit [39]. In recent years, yoga and meditation practices have become an increasingly popular and accepted practice in the United States. The health benefits of yoga practice are widespread. Notably, yoga has been shown to reduce stress, improve blood glucose, blood pressure, and cholesterol levels as well contribute to weight loss [40,41]. In western medicine, Hatha yoga, a commonly practiced form of yoga places emphasis on the physical component of the yoga practice [40].

Sudarshan et al. investigated the effects of 12 one-hour weekly Hatha yoga sessions on anxiety, depression, range of motion, and flexibility following breast surgery. The study showed that the Hatha yoga intervention significantly improved flexibility during right and left shoulder abduction, as well as range of motion during left shoulder flexion following breast surgery-related procedure [42].

Electro-Puncture and Acupuncture

Acupuncture is a form of traditional Chinese medicine that involves the stimulation of predefined acupoints on the body in order to stimulate the central nervous system. In the most commonly used form of acupuncture, needles are inserted into the acupoints and subsequently manually manipulated by lifting or twisting the needle. Electroacupuncture is a more recently established technique involving the insertion of two needles within acupoint sites. The two needles serve as electrodes to pass an electric current. One of the major benefits of electroacupuncture is the ability to objectively and quantifiably measure the intensity of the electrical stimulation, which is not possible with the traditional form of acupuncture [43].

Bosco et al. investigated the efficacy of combined electroacupuncture and homeopathic medicine (Arnica montana and Apis mellifica) in place of opioid use in two breast surgery candidates who could not tolerate the standard medications due to liver disease. This combination provided sufficient pain relief, maintained liver function, reduced time spent in the post-surgical recovery area, and total time spent in the hospital [44].

Dilaveri et al. demonstrated that acupuncture in combination with massage resulted in decreased levels of anxiety, relaxation, nausea, fatigue, pain, and mood following breast reconstructive surgery compared to baseline. Although the benefits of this intervention were similar to the benefits of massage-only intervention in regards to fatigue, anxiety, relaxation, nausea, pain, and mood scores, the massage plus acupuncture intervention increased stress levels compared to massage alone [18].

Overall, acupuncture and electro-puncture were able to improve post-surgery outcomes and demonstrated to be helpful alternatives or adjuncts to standard treatment options.

All articles incorporated into this review are summarized in Table 1.

**Table 1:** Summary table of articles included in review

Author	Year	Type of Study	Level of Evidence	Alternative Therapy	Time of Intervention	Control	Intervention(s)	Finding
Dion [19]	2016	RCT	II	Massage & Meditation	Post-operative	Massage	Massage & guided meditation	No difference between control and intervention.
Téllez [3]	2016	RCT	II	Hypnosis & Music	Pre-operative	Standard care	(1) Music (2) Hypnosis & music	Music reduced stress and anxiety. Hypnosis plus music reduced stress, anxiety, and depression, while increasing optimism and wellbeing.
Serra-Añó [20]	2018	RCT	II	Myofascial release	Post-operative	Placebo manual lymphatic drainage	Myofascial release	Myofascial release increased shoulder movement, functionality and decreased pain perception.
Deng [23]	2021	RCT	II	Aromatherapy &/or music therapy	Pre-operative and after tracheal extubation	Standard care	(1) Aromatherapy (2) Music (3) Aromatherapy & music	Aromatherapy and music therapy each lowered pain intensity, IL-6, HMGB-1, and anxiety. Combined therapy was superior.
Franco [29]	2016	RCT	II	Aromatherapy	Pre-operative	Unscented oil	Lavender fleur oil	Lavender and unscented oil both increased positive STAI score totals, with lavender increasing them to a greater degree.
Ucuzal [17]	2014	RCT	II	Foot massage	Post-operative	Standard analgesic treatment	Foot massage & standard analgesic treatment	Foot massage decreased pain and vital signs.
Kim [28]	2006	RCT	II	Aromatherapy	Post-operative	Supplemental oxygen	Lavender oil & supplemental oxygen	No difference in narcotic requirements or objective pain scores between control and intervention. Lavender oil increased pain control satisfaction.
Shammas [30]	2021	RCT	II	Essential Oil	Pre-, peri-, and post-operative	Coconut oil	Lavender oil	No difference between control and intervention.
Sudarshan [42]	2013	Prospective Cohort	IV	Yoga	Post-operative	Assessment prior to start of yoga sessions	Hatha yoga (12 sessions)	Yoga improved physical function, anxiety, depression, and pain.
Li [26]	2011	RCT	II	Music	Post-operative	Routine nursing care	Music therapy	Music reduced the Pain Rating Index.
Soo [24]	2016	RCT	II	Music & Meditation	Peri-operative	Standard care	(1) Guided meditation (2) Music	Meditation and music reduced anxiety and fatigue. Meditation additionally decreased pain.
Kwekkeboom [36]	2001	RCT	II	Guided imagery	Post-operative	Analgesic medication only	Analgesic medication plus & guided-imagery intervention	No difference between control and guided-imagery intervention.
Montgomery [38]	2007	RCT	II	Hypnosis	Pre-operative	Nondirective empathic listening (attention control)	Hypnosis session	Hypnosis decreased analgesia use, pain intensity, nausea, discomfort, and surgical time.
Schnur [37]	2008	RCT	II	Hypnosis	Pre-operative	Attention control	Hypnosis session	Hypnosis decreased negative VAS* scores and increased relaxation.
Dilaveri [18]	2020	RCT	II	Massage & Acupuncture	Post-operative	Massage alone	Massage & acupuncture	Massage alone reduced stress to a greater degree than combined therapy.
Wren [25]	2019	RCT	II	Meditation & Music	Peri-operative	Standard care	(1) Lovingkindness meditation (2) Music	Meditation improved pain, self-care, and heart rate. Music improved pain.
Bosco [44]	2018	Case report	VI	Homeopathic medicine & electro-acupuncture	Pre- and post-operative	N/A	Homeopathic treatment (Arnica montana 15CH and Apis mellifica 15CH) & electro-acupuncture treatment	Electro-acupuncture stabilized the autonomic nervous system.

Discussion

ERAS protocols focus on reducing post-operative pain and improving the patient’s overall experience. In addition to the standard care addressed in ERAS protocols, a multitude of low-risk adjunctive options exist for increasing patient satisfaction, with the potential for creating superior outcomes. The alternative practices discussed in this review outline such benefits. However, patients are often unaware that complementary measures such as these exist and most physicians do not receive education regarding implementation of these therapies in the clinical setting. Complementary therapies can be tailed to the needs and interests of patients. For instance, patients with an interest in music may benefit more from music therapy compared with patients who lack this interest. Thus, physician and patient education are critical to ensure that patients have the empowering experience of choosing the adjunctive therapies that best fits their needs.

Alternative therapies can play a role in optimizing the surgical experience for patients undergoing breast procedures, but the intervention should be tailored to each patient. If a patient has the autonomy to select which interventions they choose to utilize based on their lifestyle and personal preferences, adherence may be improved. Most interventions are fairly easy to implement and do not add a lengthy teaching component to patient-provider encounters. Additionally, many can be self-administered and/or taught through self-help books, video recordings, and classes. Once introduced in the hospital setting, many interventions can be continued at home with little or no cost to the patient. Furthermore, alternative therapies are extremely low-risk and can be utilized in patients with contraindications to standard medications or those wishing to minimize their medication exposure.

The studies reviewed should be considered in light of limitations. One limitation in several of the papers was the small sample size which could affect the power of the studies. Further studies with a larger sample size are needed to further strengthen the established relationship between alternative therapies and the reduction of post-operative complications. An additional limitation inherent to alternative therapy interventions is lack of concealment or blinding. Given the impossibility of blinding individuals to interventions including massage, meditation, hypnosis, music, myofascial release, aromatherapy, guided imagery, and electro-puncture, patients are aware of the interventions they are receiving. This can lead to a placebo bias that may sway is review [45]. Studies have demonstrated that several neurotransmitter systems, such as opiate and dopamine systems, are involved in the placebo effect, which may explain its impact on pain control [46]. However, these interventions pose essentially no risk relative to their potential benefits. Therefore, the potential benefits of placebo effects may add to the rationale for implementing these interventions.

## Conclusions

Alternative medicine can fill a major gap in surgical recovery in the pre-, peri-, or post-surgical periods. Given the well-established psychosocial component of the pain experience, alternative medicine can play a role in reducing the perception of pain, as well as ameliorating the anxiety, depression, stress, and fatigue endured throughout the surgical experience. This review demonstrates the benefit of sole alternative medical interventions, as well as the potential synergistic benefits of combination therapies. Although most surgeons may not be aware of the efficacy, affordability, availability, and feasibility of these alternative therapies, these interventions could greatly benefit the patient experience. These numerous potential positive benefits of the discussed alternative therapies demonstrate a need for increased awareness and implementation of these approaches for both surgeons and patients to empower patients and improve the surgical experience.
